# Complications Associated with the Use of Supraglottic Airway Devices in Perioperative Medicine

**DOI:** 10.1155/2015/746560

**Published:** 2015-12-13

**Authors:** Pavel Michalek, William Donaldson, Eliska Vobrubova, Marek Hakl

**Affiliations:** ^1^Department of Anaesthesia and Intensive Medicine, 1st Medical Faculty, Charles University in Prague and General University Hospital, U Nemocnice 2, 120 21 Prague, Czech Republic; ^2^University of East Anglia, Norwich Research Park, Norwich, Norfolk NR4 7TJ, UK; ^3^Department of Anaesthetics, Antrim Area Hospital, Bush Road, Antrim BT41 4RD, UK; ^4^Department of Anaesthesia and Intensive Medicine, St. Anne University Hospital, Pekarska 53, 656 91 Brno, Czech Republic

## Abstract

Supraglottic airway devices are routinely used for airway maintenance in elective surgical procedures where aspiration is not a significant risk and also as rescue devices in difficult airway management. Some devices now have features mitigating risk of aspiration, such as drain tubes or compartments to manage regurgitated content. Despite this, the use of these device may be associated with various complications including aspiration. This review highlights the types and incidence of these complications. They include regurgitation and aspiration of gastric contents, compression of vascular structures, trauma, and nerve injury. The incidence of such complications is quite low, but as some carry with them a significant degree of morbidity the need to follow manufacturers' advice is underlined. The incidence of gastric content aspiration associated with the devices is estimated to be as low as 0.02% with perioperative regurgitation being significantly higher but underreported. Other serious, but extremely rare, complications include pharyngeal rupture, pneumomediastinum, mediastinitis, or arytenoid dislocation. Mild short-lasting adverse effects of the devices have significantly higher incidence than serious complications and involve postoperative sore throat, dysphagia, pain on swallowing, or hoarseness. Devices may have deleterious effect on cervical mucosa or vasculature depending on their cuff volume and pressure.

## 1. Introduction

**Figure 1:**
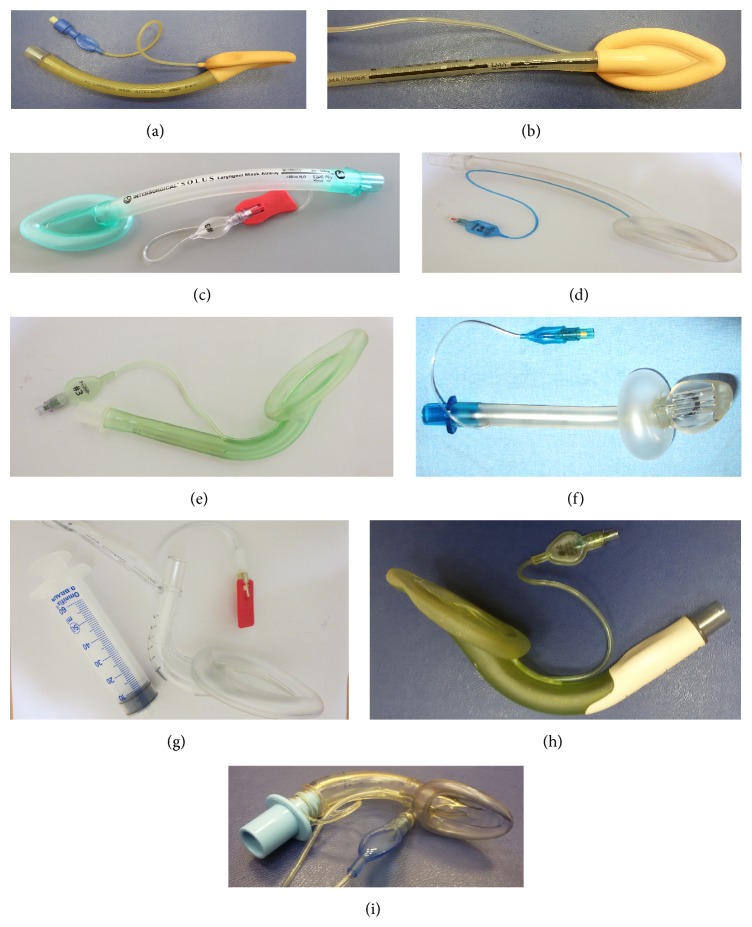
Main commercially available SGA devices without separated gastric channel (1st generation). (a) LMA Classic, (b) LMA Flexible, (c) LM Solus, (d) LM Portex Soft Seal, (e) LM AuraOnce, (f) Cobra PLA, (g) LMA Fastrach, (h) LM Aura-i, and (i) air-Q intubating laryngeal airway. Last three devices are designated as conduits for tracheal intubation.

**Figure 2:**
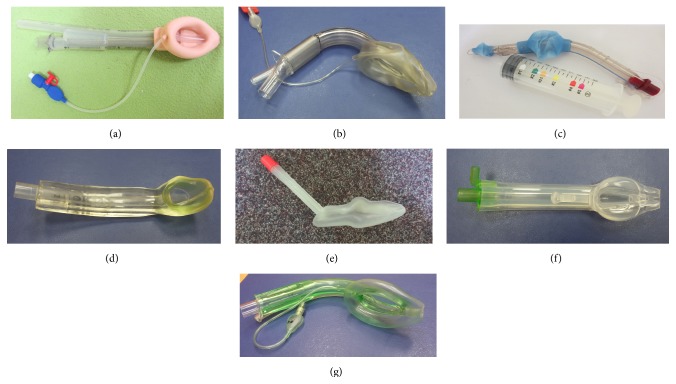
Main SGA devices with a mechanism for drainage of gastric contents (2nd generation). (a) ProSeal LMA, (b) Supreme LMA, (c) Laryngeal Tube Suction-D, (d) i-gel, (e) SLIPA, (f) Baska mask, and (g) AuraGain LM.

Supraglottic airway devices (SGAs) are tools used for airway management in anesthesia and also in certain situations outside the operating room [1]. They are less invasive than endotracheal tubes, which is attributed to their positioning outside of the larynx. Several classifications of these devices have been proposed: based on the absence or presence of a drainage channel, site and mechanism of sealing, or other features [2, 3]. The most commonly used classification divides the SGAs into 1st-generation devices containing only a breathing lumen (Figure 1) and 2nd-generation SGAs (Figure 2), which possess an additional channel for drainage of gastric contents.

Another logical classification relates the sealing site of SGAs and may be divided into base-of-tongue (BT) or pharyngeal sealers and perilaryngeal (PL) sealers [3]. Base-of-tongue sealers were invented more than 65 years ago, when Leech introduced his “pharyngeal bulb gasway” in 1937 [4]. The second line of SGAs, perilaryngeal sealers, was derived from the classical laryngeal mask airway (LMA Classic, cLMA), invented by Brain and patented in the UK in 1982 (GB2111394A) [5]. Various devices, described in Table 1, have been invented and introduced into clinical practice since 1992. Modern SGAs are disposable, withstand high seal pressures, are easy to insert with a high success rate more than 95%, and possess a mechanism for separation of respiratory and gastrointestinal tracts [4].

Initially, SGAs were used mainly for maintenance of a patent airway during elective procedures under general anesthesia but, during years following the release of the prototypical cLMA, these devices have also found other areas of utilization, for example, as conduits for tracheal intubation in difficult laryngoscopy scenarios [6] or as airway adjuncts in cardiac arrest or in prehospital medicine [7]. Several review articles have focused on individual devices and particular clinical indications for their use but none has been targeted specifically at complications associated with their insertion.

## 2. Complications

The use of supraglottic airway devices (SGAs) in perioperative medicine is now widespread. The 4th National Audit Project (NAP4), which was conducted in the United Kingdom, estimated that 56% of general anaesthetics performed were carried out using SGAs to manage the airway [8]. This project, led by the Royal College of Anaesthetists, looked into complications of airway management in general in the United Kingdom. In all, 33 of the events that were reported to NAP4 involved SGAs [9]. These events included aspiration, airway trauma, loss of the airway on insertion, failed insertion, displacement after insertion, loss of airway during maintenance, and extubation-related problems. In most cases, multiple factors such as obesity, comorbidities, traumatic insertion, inappropriate use of the devices, low operator experience, nonstandard patient positioning, or shallow anesthesia contributed significantly to these complications.

Cheon et al. found that the overall incidence of complications depends on a patient's body mass index (BMI) and also on their age—obese patients with a BMI over 30 kg·m^−2^ and those older than 46 years have a significantly higher chance of developing difficulties with ventilation and intraoperative laryngospasm [10].

Most reports dealing with the complications associated with the SGAs come from their use in elective procedures. However, the SGAs are also integral part of difficult airway management and recommended back-up plan in failed intubation according to the guidelines of various societies (Difficult Airway Society, American Society of Anesthesiologist, French National Society of Anesthesiology). These scenarios involve emergency procedures in nonfasted patients and in those with significantly increased risk for aspiration of gastric contents and therefore the incidence of complications should be theoretically multiplied to the elective use of these devices. Nevertheless, any of large cohorts describing the use of ILMA [11] or other SGAs [12] in difficult airway patients did not look specifically at the complication rate.

Complications discussed in this paper include those with serious sequelae such as aspiration of gastric contents, trauma, nerve injuries, and compression of vascular structures and also minor adverse effects such as hoarseness, sore throat, or swallowing difficulties.

### 2.1. Aspiration of Gastric Contents

Regurgitation of gastric contents is a process that can occur under anesthesia and which may lead to pulmonary aspiration. Pulmonary aspiration of gastric content can be defined as the inhalation of material into the airway below the level of the vocal cords. Depending on the nature, volume, and pH of the material inhaled patients can suffer morbidity and even mortality. The prevention of aspiration is one of the hallmarks of safe practice in anesthesia.

The incidence of regurgitation under anaesthesia is unknown but the incidence of pulmonary aspiration has been described as between 0.01 and 0.06% in general [14]. Aspiration during anesthesia accounts for between 2.6% and 3.5% of cases in surveillance studies and closed claims analyses [15, 16] with no such claims relating to aspiration during LMA anesthesia [17]. NAP4 featured aspiration as the primary event in 17% of cases and was the commonest etiology for death and brain damage [9].

#### 2.1.1. Aspiration and the 1st-Generation Perilaryngeal Sealers

The LMA Classic (cLMA) is the most studied SGA with over 2500 publications. A publication resulting from evaluation of one of the prototypes noted that there were no signs of regurgitation in 100 patients [18].

The first published case of significant aspiration leading to pneumonia was reported in 1990 [19]. This prompted a series of similar cases [20]. In reply to Nanji and Maltby's case report and an accompanying editorial, Dr. Brain—the inventor of the cLMA—pointed out that the patient described in the case report was unsuitable for use of the cLMA and also highlighted tips for reducing the risk of regurgitation, recognizing the problem and a treatment algorithm [21].

A large meta-analysis of publications describing aspiration and the cLMA by Brimacombe reported that the incidence of pulmonary aspiration with the cLMA was 2.3 per 10000 cases, which was comparable to the rates with endotracheal intubation or facemask anesthesia [22]. Two large studies also report low rates of aspiration with the device: 1 case in 11910 patients [23] and 3 cases in 35620 patients in a study which showed a similar incidence when compared with endotracheal intubation [24].

The mortality associated with aspiration and anesthesia is estimated to be 5% [25, 26]. Despite this, before completion of NAP4, only two deaths had been reported following aspiration with a cLMA [9, 27, 28].

Overall, the risk of aspiration for the cLMA is low and comparable to that seen with anesthesia using other devices to maintain the airway. No relevant data is available for the other laryngeal masks of the 1st generation such as the LMA Flexible, Intubating LMA Fastrach, AuraOnce, Aura-i, and La Premiere.

#### 2.1.2. Aspiration and the 1st-Generation Base-of-Tongue (Pharyngeal) Sealers

These devices include the Laryngeal Tube (King LT) and Cobra Perilaryngeal Airway. Other devices from this group, Combitube and Easy Tube, are used mainly in prehospital medicine. Miller and Light suggested that the storage capacity of the Laryngeal Tube for regurgitated gastric contents inside the pharynx is higher than in cLMA, which may decrease the risk for aspiration with this device [29]. The safety of another SGA from this group (Cobra PLA) was questioned in a report by Cook and Lowe—they had to halt their study after two cases of aspiration (with an incidence of 6.9%) [30]. They highlighted that the device does not possess any mechanism for aspiration protection or obstruction of the esophagus. One of these two aspirations of gastric contents was reported during rotation of a malpositioned device during laparoscopic surgery.

#### 2.1.3. Aspiration and the 2nd-Generation Devices

These devices fall into two subcategories: devices with a dedicated second gastric channel (LMA ProSeal (PLMA), LMA Supreme (SLMA), Laryngeal Tube Suction II (LTS II), i-gel, Baska Mask, AuraGain, and 3gLM) and devices designed to trap and store regurgitated material (SLIPA).

The PLMA has been described in several cases where it served to protect the airway from regurgitated content [31]. Despite this, there exist a number of cases describing aspiration associated with use of the device [32–34].

The ProSeal requires careful positioning in order to function correctly, and if this is not the case then the device may actually increase the risk of regurgitation by contributing to gastric insufflation [33]. It is recommended that the position should be checked by following the manufacturer's recommendations or by the passage of a gastric tube. Novel techniques have been described for insertion using the gum-elastic bougie [35], gastric tubes [36], and suction catheters [37]. Less experienced users may benefit from the gastric tube-guided insertion of the PLMA [38].

The Laryngeal Mask Airway Supreme (SLMA) is a newer device with very little evidence of regurgitation associated with its use and no described cases of aspiration. A recent meta-analysis showed very low incidence of complications [39] and a large observational study of 700 patients undergoing caesarean section found no cases of aspiration [40].

The i-gel differs from the devices described above in that it has a cuff that does not require inflation. The i-gel also possesses a separate gastric channel. Despite, and in some cases because of, these features there have been cases described in the literature of both regurgitation and aspiration of gastric contents: Gibbison et al. described a case series of three patients who regurgitated under anesthesia [41]. In two of these cases, the authors stated that the i-gel protected the patients from aspiration. The third case did aspirate, but with no complications, and was discharged the same day. The authors state that the drain tube allowed recognition of regurgitation, which they suggest may have gone undiscovered with the use of a first-generation device. They conclude that the incidence of regurgitation and aspiration for the device is low and noted that—at the time—no patients appeared to have come to harm from such episodes. This phenomenon of “recognition of regurgitation” is also described in a case by Liew et al. [42].

The i-gel has been found to have a lower esophageal seal than both the cLMA and PLMA, but together with the PLMA it was found to drain away regurgitated fluid effectively [43]. The lower esophageal seal is likely due to the fact that the tip of the i-gel is narrower—which was a deliberate design intended to decrease dysphagia associated with SGAs [3].

The SLIPA, LTS-II, and its disposable version LTS-D have no published reports referring to either regurgitation or aspiration.

There is still a lack of high-quality evidence associated with those SGAs with an incorporated gastric channel with regard to their ability to deal with the risk of regurgitation and aspiration and large, well-conducted trials are needed in this area. Despite this lack of evidence, the authors of NAP4 made recommendations regarding the use of 2nd-generation SGAs, including the following: “If tracheal intubation is not considered to be indicated but there is some (small) increased concern about regurgitation risk a second generation supraglottic airway is a more logical choice than a first generation one.” Similar recommendation has been also published in a recent editorial [44].

### 2.2. Trauma

Microscopic trauma associated with insertion of SGAs is thought to be relatively common but of little consequence and, in any case, difficult to detect. Macroscopic trauma, however, may lead to significant morbidity. It may occur at a number of sites and be caused by a number of mechanisms (Table 2). The main areas are the lips, teeth, pharyngeal mucosa, tongue, uvula, epiglottis, and the laryngeal apparatus [45]. Trauma may be caused directly by forceful placement or indirectly by compression and can result in laceration and bleeding, ischemic injuries, or neurological injuries as a result of compression of nerves [46], which will be discussed separately.

#### 2.2.1. Minor Trauma

Dental injuries occur in about 1% of anesthetics and make up a significant proportion of legal claims against practitioners [47]. Dental injuries occur less frequently with SGA insertion than they do with direct laryngoscopy [48], but they may be also associated with removal of these devices.

There is only one publication mentioning dental damage in association with either the cLMA [49] or intubating LMA [50]. Few studies have looked for or mentioned dental damage in association with the i-gel but the incidence was almost zero [51–53].

The cLMA has been reported as the victim of trauma in one report: a sharp crown exposed by decay tore the cuff of two devices during insertion [54].

The presence of blood on the device upon removal of a SGA often indicates minor trauma associated with device insertion. The reported incidence of this for the cLMA is between 12 and 15% [55] and 9 and 22% in association with the PLMA [56, 57], depending on insertion technique. An incidence of blood staining of 20% has been described with the Guardian CPV laryngeal mask [57]. The typical incidence of blood on the i-gel at removal is between 4% and 13% [58–60] but has been reported to be as high as 20%, albeit in novice users [61]. The AuraOnce laryngeal mask was associated with a very low (2%) incidence of blood staining after its removal [62] but reached 10% in another study [63]. The presence of blood on the Cobra airway may be as high as 50% [64]. Aydogmus et al. reported a 7.5% incidence of blood staining on the LMA Supreme in pediatric patients, which was significantly lower than with the LMA ProSeal [65]. Insertion of the SLIPA may be associated with minor trauma in more than 20% of patients [66, 67]. Insertion of a novel SGA, the Baska mask, has been associated with significantly higher incidence of oropharyngeal trauma than the single-use cLMA, as reported by blood staining observed on the device after removal. However, this fact was not associated with an increased incidence of laryngospasm or postoperative complaints [68]. Five different 2nd-generation SGAs were inserted by inexperienced operators in another study [69]. SLMA, PLMA, i-gel, and LTS-D showed lower incidence of blood staining on removal than SLIPA. However, their patients were not surveyed postoperatively for symptoms of pharyngolaryngeal morbidity.

Theiler et al. analyzed complications associated with the use of i-gel in 2049 patients. They experienced 1.2% incidence of laryngospasm, 3.9% incidence of blood staining on the device, 2 cases of transient nerve damage, and one case of glottic hematoma after uncomplicated device insertion [70].

Injury to the lingual frenulum during insertion has been reported with use of the LMA ProSeal [71, 72] and the i-gel [73]. The mechanism of injury is usually backward folding of the tongue on insertion [74], thus stretching the lingual frenulum.

Trauma to the uvula or uvulitis has been described following insertion of laryngeal mask airway [75].

Ischemia of the tongue has been described in association with the intubating LMA after prolonged insertion [76] and also with the cLMA—again after a period of prolonged insertion [77]. A vacuum-like effect has been suggested to cause a hematoma on the lateral edge of the tongue following insertion of the 3gLM airway [78].

Pharyngeal lacerations have also been reported in association with the cLMA and in one case this led to the pulmonary aspiration of blood [79]. A different site of injury (aryepiglottic fold) led to massive hemorrhage after withdrawal of an i-gel [80].

Arytenoid dislocation has been reported after airway maintenance with a cLMA [81] which could be caused by direct contact with arytenoids, insertion with inflated cuff, or device rotation during placement. Despite strictly recommended methods of insertion, both uvular [82, 83] and epiglottic injuries [84] have been associated with use of the laryngeal mask. Arytenoid cartilage dislocation, as well as recurrent laryngeal nerve trauma with subsequent unilateral vocal cord palsy, has been described in association with the SLIPA [85]. Both complications led to persistent hoarseness.

#### 2.2.2. Major Trauma

Severe damage to the pharyngeal structures or esophagus leading to life-threatening complications is extremely rare with SGAs. However, a few cases have been described in the literature. Blind insertion of a tracheal tube through the intubating laryngeal mask airway (ILMA) probably caused perforation of the esophageal diverticulum in an elderly patient which led to development of a pneumomediastinum [86]. The patient died nine weeks later due to multiorgan failure. Deep neck abscess and mediastinitis following pharyngeal perforation caused by cLMA insertion have been described in a low-risk elective procedure [87]. A similar complication causing a prolonged ICU and hospital stay was described following traumatic cLMA insertion for an elective urology procedure [88]. Both patients survived but required thoracic surgery intervention and prolonged mechanical ventilation. A posterolateral lesion of the pharyngeal wall after an uncomplicated insertion of cLMA was described in another elective patient [89]. Subsequent subcutaneous emphysema, pneumomediastinum, and pneumoperitoneum resolved spontaneously after several days. A recent report presented serious oropharyngeal trauma associated with the use of i-gel [90]. An elderly patient with multiple osteophytes on the cervical spine developed an airway obstruction few weeks after the procedure. Extensive hypopharyngeal mucosal erosions with denudation of the cricoid cartilage and subsequent supraglottic edema resulted in emergency tracheotomy and prolonged artificial ventilation. The authors suggested that age, duration of surgery, and pathology of the cervical spine contributed to this trauma.

### 2.3. Nerve Injuries

Innervation of the structures which SGAs come into contact with is complex. There are risks associated with device insertion and fixation and with the device in situ. Lesions to the lingual nerve have been repeatedly described with use of the cLMA [91–93], the PLMA [94], SLMA [95, 96], and i-gel [95, 97]. Injuries to the hypoglossal nerve have been described in association with using the cLMA [98, 99], PLMA [100], and SLMA [101]. Injuries to the recurrent laryngeal nerve have been described in association with the cLMA in adults [102–105] or children [106] and with insertion of the SLIPA [85].

Whilst the etiology of neurological injury by SGAs is multifactorial, in many of these cases the inflatable cuff of the devices was implicated—either by causing the device to be too rigid during insertion or by direct compression of nervous structures whilst the device was in place.

Despite its lack of a cuff, nerve injury in association with the i-gel has been described; Theron described a case of likely mental nerve injury [107]. In a reply to this letter, Chapman stresses the importance of taping the device correctly and of correct size selection, lubrication, and insertion technique [108].

Renes presented a case of bilateral lingual nerve injury in association with the use of an i-gel [97]. Another letter also refers to symptoms, which are consistent with an injury to the lingual nerve [73].

In their cohort study, Theiler et al. reported two instances of neurological damage [70]. The authors emphasise that device selection should involve choosing the smallest device that provides an adequate airway seal—particularly in those patients who are overweight or who are anesthetized for longer procedures.

### 2.4. Minor Complications

These mainly include sore throat, swallowing difficulties, and hoarseness lasting for up to several days after anesthesia. The etiology of postoperative sore throat (POST) is unclear. Factors associated with its increased incidence include female sex, use of suxamethonium, younger patients, and patients undergoing gynecological surgery [109]. Trauma to different areas by different devices (SGAs and endotracheal tubes) causes a similar incidence of sore throat postoperatively [110].

The incidence of sore throat associated with use of the cLMA ranges from 5.8% to 34% compared with 14.4% to 53% in association with endotracheal intubation [109]. There are differences in the sites of forces applied by a supraglottic airway (posterior pharynx) and endotracheal tubes (glottic entrance) which explain the different nature of complaints associated with them; dysphonia is more common with an endotracheal tube, and dysphagia more common with SGAs [111]. The incidence of sore throat after the use of other SGAs is not very different—AuraOnce LM up to 22%, the i-gel between 5% and 17% [62, 112]. Kihara et al. do not recommend using ILMA instead of cLMA for routine procedures due to its significantly higher pharyngolaryngeal morbidity including sore throat (34–59%) and swallowing difficulties (up to 31%) [113]. Limited evidence is available to show that those SGAs with a gastric channel (2nd generation) may cause less sore throat and swallowing difficulties than the 1st-generation devices [112]. SLIPA has demonstrated a very low incidence (2%–8.6%) of postoperative sore throat and swallowing difficulties [66, 67]. The incidence of minor postoperative complaints has also been studied in other base-of-tongue sealing devices. The Cobra PLA airway may cause sore throat postoperatively with the incidence rising up to 31% as the cuff volume and pressure are increased [114]. Turan and colleagues found a significantly higher incidence of POST in patients managed with the Cobra PLA airway—50%—compared to those who had the PLMA or Laryngeal Tube inserted [64]. The incidence of sore throat and dysphagia following insertion of the LT or LTS II (LTS-D) has been reported at between 8% and 20% [115, 116]. The LTS-D showed a significantly higher incidence of postoperative sore throat and dysphagia than both the i-gel and SLMA [117].

There are several factors that may lead to the development of a sore throat with SGA use and they are highlighted in Table 3.

## 3. Effect of SGAs on Cervical Vascular Structures

Supraglottic airway devices may cause distortion of anatomical structures in the neck. The inflated cuff of laryngeal mask airways lies at the level of the cricoid cartilage and its expansion may change the position and/or diameter of the common carotid artery and internal jugular vein.

The clinical effects of cuff inflation on neck vessels were first studied by Colbert et al. [118]. They initially performed a pilot evaluation of carotid artery diameter and flow in a patient who was scheduled for elective surgery under general anesthesia. The cross-sectional area of both carotid arteries significantly decreased after inflation of the LMA cuff which was compensated for by an increase in flow velocity and carotid blood flow. In their subsequent study, the authors evaluated carotid artery hemodynamics in seventeen patients who had cLMA inserted for routine elective cases under general anesthesia [119]. The cross-sectional area of the carotid arteries significantly differed between cuff inflation and deflation. Carotid blood flow was also significantly lower during cuff inflation whereas no difference was observed in flow velocity. Reduction in the carotid artery diameter was more marked in patients older than 60 years where the cross-sectional area dropped after inflation by more than 60% when compared with the area measured during cuff deflation. The results of this study suggest a potential deleterious effect of the laryngeal mask airway on brain perfusion in older patients, which can be further potentiated by a presence of sclerotic plaques inside the carotid arteries.

The significance of these findings in patients with normal perfusion parameters remains debatable. Compression of neck vessels may have deleterious effects on brain perfusion in patients with low-flow conditions, such as resuscitation in cardiac arrest or hypovolemia. Segal et al. studied the relationship between three SGAs (King Laryngeal Tube Suction-D, Laryngeal Mask Airway Flexible, and Combitube 41F) and carotid artery blood flow in an experimental swine model of cardiac arrest [120]. The authors found that insertion and cuff inflation of each of the three SGAs caused a significant reduction in carotid blood flow as compared with the control group, which was managed with tracheal intubation. Postmortem arteriograms were performed for each airway device and showed that all three SGAs were associated with a compression of the common, internal, and external carotid arteries.

Laryngeal mask insertion may change the anatomical relationship of the common carotid artery and internal jugular vein [121]. This changed in 8.3% of children following inflation of the laryngeal mask cuff [122]. Intracuff pressures of the LMA should be measured regularly during general anesthesia because an overinflated cuff may cause congestion of the neck veins [123].

There is no evidence available regarding the effect of other or newer SGAs such as the SLMA, i-gel, SLIPA, Cobra PLA, or Laryngeal Tubes on carotid cross-sectional area or carotid blood flow.

## 4. Pressures Exerted by SGAs on Pharyngeal Mucosa

Tracheal tubes may cause damage to the tracheal mucosa which can manifest itself as postintubation edema, narrowing or, in prolonged intubation, as tracheal stenosis [124]. The inflated cuff of the tracheal tube may also damage the recurrent laryngeal nerves, more commonly in children [125]. Supraglottic airway devices do not have any effect on the tracheal mucosa. Marjot raised the first concerns about a negative effect of SGAs on oropharyngeal mucosa in 1993 [126]. He measured the intracuff pressures inside the bowl of the cLMA in ten patients under general anesthesia and found them to range between 103 and 251 mmHg. He suggested that transmitted mucosal pressures might potentially exceed capillary perfusion pressure in the hypopharynx. Similar concerns were also raised by O'Kelly and colleagues [127].

Subsequent studies were performed by Keller and Brimacombe's group. These researchers put microchip sensors on the outer surface of various SGAs and measured the pressures exerted by these devices on various parts of pharyngeal and perilaryngeal areas. The findings of their initial studies suggested that the actual pressures are probably much lower than those calculated and do not exceed the capillary perfusion pressures [128].

The same authors showed, on a cadaver model, that pressures exerted by the tracheoesophageal Combitube on pharyngeal and esophageal mucosa are quite high and that they may exceed mucosal perfusion pressures [129]. Another type of base-of-tongue sealer, the Laryngeal Tube, also showed a potential for pressure trauma to pharyngeal structures [130]. Extended insertion of supraglottic airway devices may significantly contribute to the pharyngeal mucosa hypoperfusion. LMA ProSeal inserted over a period of 12 hours was associated with a significantly increased incidence of mucosal injury in an animal model when compared with shorter periods of time [131]. Nitrous oxide, which is still used by some anesthesiologists, diffuses into the cuff of any inflatable SGA, expanding its size and increasing the intracuff pressures [132]. However, these higher pressures caused only mild histological signs of pharyngeal mucosal injury in an animal model for procedures of up to 2 h of duration [132, 133].

Human studies have been carried out for most currently used SGAs. The cLMA was compared with the intubating LMA in anesthetized and paralyzed adults. The intubating LMA was associated with significantly higher seal pressures but pressures exerted on the mucosa in the distal oropharynx were more than 157 cm H_2_O, exceeding mucosal perfusion pressures in that area [134]. Pressures exerted on the pharyngeal mucosa with the intubating LMA were even higher than in devices employing base-of-tongue or pharyngeal sealing as their primary mechanism (Laryngeal Tube, Easy Tube, or Combitube) [135]. The i-gel airway and LMA Supreme were compared in regard to pressures exerted onto the oropharyngeal and perilaryngeal mucosal tissue [59]. Both devices exhibited very low pressures (not exceeding 10 cmH_2_O). The i-gel did not show any pressure differences but pressures exerted by LMA Supreme were lower at the base of the tongue and distal oropharynx than in the hypopharynx. No data about their effect on mucosa are available for the SLIPA, Cobra airway, or novel devices such as the Baska mask, AuraGain LM, Guardian LM, or 3gLM.

Two studies confirmed an increase in the cuff volume, intracuff pressures, and transmitted mucosal pressures, depending on the increasing altitude, in tracheal tubes and SGAs when cuffs were filled with air [136, 137]. These findings raised concerns as whether to fill these cuffs with saline, to check the intracuff pressures at regular intervals, or to use SGAs with a noninflatable cuff such as the i-gel [138].

## 5. Conclusions

In many indications, such as for elective procedures outside of the thorax and abdomen in patients without increased risk for gastric content aspiration, SGAs have already replaced tracheal intubation. These devices are still developing in order to overcome their limitations and to minimize the incidence of complications or minor adverse events associated with their insertion.

Complications associated with the correct use of the SGAs are relatively rare and most of them are not life-threatening. They are often associated with a deviation from the manufacturers' advice on usage of their devices. Aspiration remains a problem, which can have serious and even fatal consequences. Its incidence is extremely low, comparable with the incidence of aspiration in tracheal tube anesthesia [25]; however, its real occurrence may be underreported [9]. Although there is some limited evidence that newer devices with an additional gastric channel may offer greater protection from regurgitation and aspiration this still requires robust studies to be carried out. Assessment of the risk of aspiration is a key component of the preanesthetic evaluation and should be used to guide device selection.

Nerve injuries may be avoided by careful insertion and by limiting cuff inflation pressure in accordance with advice from the manufacturer. Limiting cuff pressures may also decrease the incidence of sore throat.

The effects of SGAs on cervical vascular structures and microcirculation of the pharyngeal mucosa are not yet completely explored. It appears that negative effects are directly related to cuff volume and its internal pressure.

## Figures and Tables

**Table 1:** Main commercially available SGAs divided into the devices without and with aspiration protection mechanism and according to the sealing mechanism [4], (I)—may be used as a conduit for an insertion of tracheal tube. LTS-D: Laryngeal Tube Suction device, PLA: perilaryngeal airway, LMA: laryngeal mask airway, LM: laryngeal mask, ILA: intubating laryngeal airway, and SLIPA: Streamlined Liner of Pharyngeal Airway. SALT: Supraglottic Airway Laryngopharyngeal Tube.

Aspiration protection	Base-of-tongue (BT) sealers	Perilaryngeal (PL) sealers
None (1st generation)	VBM Laryngeal Tube (VBM, Germany) King Laryngeal Tube (King System, USA) Cobra PLA (Pulmodyne, USA) Cobra Plus (Pulmodyne, USA)	LMA Classic (LMA Co., Seychelles) LMA Unique (LMA Co., Seychelles) LMA Flexible (LMA Co., Seychelles) LMA Classic Excel (I) (LMA Co., Seychelles) AuraOnce LM (Ambu, Denmark) Aura-i LM (I) (Ambu, Denmark) Portex Soft Seal (Smith Med., UK) Solus LM (Intersurgical, UK) Sheridan LM (Teleflex, USA) La Premiere Plus LM, LaEncore Plus LM (Armstrong Medical, UK) Vital Seal LM (GE Healthcare, USA) Ultra CPV (AES, USA) Intubating LMA, Fastrach (I) (LMA Co., Seychelles) CTrach LMA (I) (LMA Co., Singapore) Air-Q ILA (I) (Mercury Medical, USA)

Gastric channel or storage container (2nd generation)	Combitube (Covidien, USA) Rusch Easy Tube (Teleflex, USA) VBM LTS II (VBM, Germany) King LTS-D (King System, USA) SLIPA (CurveAir, UK) SALT (I) (Ecolab, USA)	LMA ProSeal (LMA Co., Seychelles) LMA Supreme (LMA Co., Seychelles) i-gel (I) (Intersurgical, UK) Aura Gain LM (I) (Ambu, Denmark) Guardian LMA (Ultimate Medical, Australia)

Gastric channel + self-energizing mechanism of seal		Baska mask (I) (Logikal Health Products, Australia) 3gLM (I) (CurveAir, UK)

**Table 2:** Sites, types, and mechanisms of traumatic injuries caused by SGAs (modified from Michalek and Donaldson [13]).

Site of injury	Type(s) of injury	Mechanism(s) of injury
Pharyngeal mucosa	Laceration Bruising	Forceful insertion, inadequate lubrication Prolonged insertion, too high cuff pressures

Laryngeal apparatus	Arytenoid dislocation Recurrent laryngeal nerve injury	Direct trauma Compression of the nerve in piriform fossa

Uvula	Trauma leading to ischemia and necrosis	Direct trauma Prolonged compression

Epiglottis	Bruising Laceration	Incorrect or forceful insertion, anatomical abnormalities

Tongue	Frenular injury Lingual nerve injury	Incorrect or forceful insertion Compression of inferior or lateral surface of the tongue by cuff or tube of SGA

Teeth	Displacement Fracture of roots	Direct trauma Biting on SGA/bite block

Lips	Laceration Nerve injury	Direct trauma Compression by device, taping to device

**Table 3:** Possible factors implicated in the development of postoperative sore throat with SGAs.

Factor	Mechanism
Insertion technique	Leading edge of deflated cuff may cause trauma Inflated cuff causes more epiglottic downfolding, which increases POST Repeated attempts are associated with increased POST

Size of device	Smaller sizes of SGAs are associated with less POST

Use of lubricants	Adequate lubrication is essential Lidocaine gel is associated with an increase in POST

Overinflation of the cuff	Some studies have shown decreased POST with intracuff pressure monitoring

Duration of surgery	Increased POST in operations of over 60 min duration

Airway gases	Lack of humidification can dry mucosal surfaces and increase POST

## Abbreviations

BMI: Body mass index
ILMA: Intubating laryngeal mask airway
LM: Laryngeal mask
LMA: Laryngeal mask airway
LTS: Laryngeal Tube Suction
NAP: National Audit Project
PLA: Perilaryngeal airway
POST: Postoperative sore throat
SGA: Supraglottic airway device
SLIPA: Streamlined liner of the pharyngeal airway.
